# Distinct prognostic value of different portal hypertension-associated features in patients with primary biliary cholangitis

**DOI:** 10.1007/s00535-021-01839-3

**Published:** 2021-12-11

**Authors:** Lukas Burghart, Emina Halilbasic, Philipp Schwabl, Benedikt Simbrunner, Albert Friedrich Stättermayer, Oleksandr Petrenko, Bernhard Scheiner, David Bauer, Matthias Pinter, Kaan Boztug, Mattias Mandorfer, Michael Trauner, Thomas Reiberger

**Affiliations:** 1 Division of Gastroenterology and Hepatology, Department of Internal Medicine III, Medical University of Vienna, Spitalgasse 23, 1090 Vienna, Austria; 2 Vienna Hepatic Hemodynamic Laboratory, Division of Gastroenterology and Hepatology, Deparment of Internal Medicine III, Medical University of Vienna, Vienna, Austria; 3 RALID Center of the ERN Rare Liver, Vienna General Hospital and Medical University of Vienna, Vienna, Austria; 4 Christian-Doppler Laboratory for Portal Hypertension and Liver Fibrosis, Medical University of Vienna, Vienna, Austria; 5 Ludwig Boltzmann Institute for Rare and Undiagnosed Diseases (LBI-RUD), Vienna, Austria; 6 CeMM Research Center for Molecular Medicine of the Austrian Academy of Sciences, Vienna, Austria

**Keywords:** Portal hypertension, PBC, CSPH, Elastography, Decompensation

## Abstract

**Background:**

Primary biliary cholangitis (PBC) may progress to cirrhosis and clinically significant portal hypertension (CSPH). This study assesses different features of CSPH and their distinct prognostic impact regarding decompensation and survival in patients with PBC.

**Methods:**

Patients with PBC were identified during a database query of our digital patient reporting system.

**Results:**

A total of 333 PBC patients (mean age 54.3 years, 86.8% females, median follow-up 5.8 years) were retrospectively assessed and 127 (38.1%) showed features of CSPH: 63 (18.9%) developed varices, 98 (29.4%) splenomegaly, 62 (18.6%) ascites and 20 (15.7%) experienced acute variceal bleeding. Splenomegaly, portosystemic collaterals and esophageal varices were associated with an increased 5-year (5Y) risk of decompensation (15.0%, 17.8% and 20.9%, respectively). Patients without advanced chronic liver disease (ACLD) had a similar 5Y-transplant free survival (TFS) (96.6%) compared to patients with compensated ACLD (cACLD) but without CSPH (96.9%). On the contrary, PBC patients with cACLD and CSPH (57.4%) or decompensated ACLD (dACLD) (36.4%) had significantly decreased 5Y survival rates. The combination of LSM < 15 kPa and platelets ≥ 150G/L indicated a negligible risk for decompensation (5Y 0.0%) and for mortality (5Y 0.0%). Overall, 44 (13.2%) patients died, with 18 (40.9%) deaths attributed to CSPH-related complications.

**Conclusion:**

In PBC, features of CSPH may occur early and indicate an increased risk for subsequent decompensation and mortality. Hence, regular screening and on-time treatment for CSPH is crucial. Combining LSM and platelets serves as a valuable preliminary assessment, as LSM < 15 kPa and platelets ≥ 150G/L indicate an excellent long-term outcome.

**Supplementary Information:**

The online version contains supplementary material available at 10.1007/s00535-021-01839-3.

## Introduction

Primary biliary cholangitis (PBC) is a rare cholestatic liver disease that may progress to cirrhosis [1, 2]. Previous studies reported that only a small number of patients showed clinically significant portal hypertension (CSPH) at the time of PBC diagnosis [3–5]. A recent study, however, observed that the 10 year cumulative incidence of CSPH is as high as 40% [5]. Since CSPH drives severe complications [6], such as variceal bleeding and development of ascites, it is of utmost clinical importance to screen for CSPH, as it impacts on prognosis and causes an increased mortality in patients with cirrhosis [7].

Only a few studies have described the prevalence and the specific manifestations of CSPH in patients with PBC so far [5, 8–11]. However, most studies either had a small sample size or did not investigate the entire spectrum of CSPH-related features and complications. Harms et al. found that 278 patients with PBC developed CSPH out of a cohort of 3224. According to their results ascites was the most prevalent feature of CSPH, accounting for 63% (*N *= 175) of all patients diagnosed with CSPH. Esophageal varices occurred less frequently, affecting only 23% (*N *= 65) [1]. Unfortunately, data concerning further disease progression after CSPH onset was not available [1].

In terms of novel and effective PBC treatments for patients with non-response/intolerance to UDCA or with high-risk of disease progression [12], the reduction of CSPH-associated complications represents a clinically relevant endpoint for future studies [13, 14].

Hence, our study aims to investigate the prevalence and features of CSPH in patients with PBC, to report the incidence of CSPH-related complications during follow-up and to assess the predictive value of different clinical characteristics regarding subsequent decompensation and survival.

## Patients and methods

### Study population (Fig. 1)

**Fig. 1:**
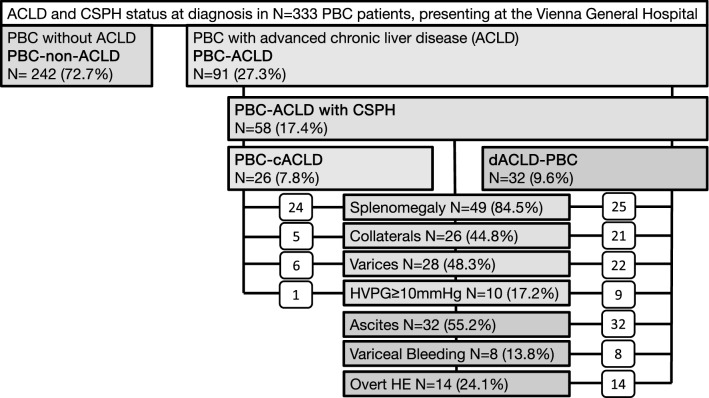
Patient flowchart

Patients presenting with suspected with PBC at the Vienna General Hospital were identified by a query of existing databases and considered for this study if meeting  diagnostic PBC criteria [2]. All patients had elevated cholestasis parameters (gGT, AP, bilirubin) as well as positive PBC-specific serology (AMA-M2; ANA-SP100; ANA-GP210, *N *= 306) and/or PBC-specific histologic features on liver biopsy (*N *= 175), thus fulfilling at least two out of three diagnostic criteria [2]. After excluding 34 patients due to mechanical cholestasis [2], the diagnosis of PBC was confirmed by clinical documentation in a total number of *N *= 333 patients.

### Study parameters

Demographic data and important aspects of our patients’ medical history were obtained from a database query of the electronic patient record system at the Vienna General Hospital (AKH Wien). Reports on radiologic imaging studies, such as CT, MRI and ultrasound were searched for portosystemic collaterals, portal vein thrombosis, features of mechanical cholestasis, biliary obstruction, such as cholelithiasis, as well as splenomegaly (> 11 cm) [15]. Trained radiologists assessed the spleen diameter by measuring the maximum distance between the inferior and the superior pole in the respective imaging modality. Information regarding presence of gastroesophageal varices and endoscopic interventions on varices were obtained from endoscopy reports. Results of liver stiffness measurements (LSM) and of hepatic venous pressure gradient (HVPG) were accessed from the patients’ electronic medical history.

### Definition of CSPH and compensated versus decompensated ACLD

CSPH was defined by presence of at least one of the following criteria: (i) gastroesophageal varices, (ii) splenomegaly > 11 cm, (iii) portosystemic collaterals; (iv) hepatic venous pressure gradient (HVPG) ≥ 10 mmHg; (v) ascites (excluding non-hepatic causes), (vi) variceal bleeding, (vii) hepatic encephalopathy and or (viii) death due to portal hypertension. We decided to include (i) gastroesophageal varices as well as (iii) portosystemic collaterals as distinct parameters for CSPH, since they require different diagnostic modalities to be detected.

Advanced chronic liver disease was defined by at least one of the following criteria: (i) liver histology showing F3/F4 fibrosis, (ii) LSM ≥ 15 kPa, (iii) thrombocytopenia (< 150 G/L), (iv) HVPG ≥ 6 mmHg and/or (v) presence of CSPH-features (as described above). Patients with compensated advanced chronic liver disease (cACLD) were characterized by at least one feature of ACLD and the absence of any previous or current decompensating events [16–18]. Patients with decompensated ACLD (i.e. dACLD) presented at least one of the following characteristics: ascites, variceal bleeding, overt hepatic encephalopathy or death caused by portal hypertension [16–18]. HVPG- and LSM-measurements were performed when clinically indicated, as previously described [19–21]. Presence and size of gastroesophageal varices was recorded according to Austrian Billroth III guidelines [22].

Considering the clinical status at baseline and within the first year of follow-up we divided our population into the following groups: (i) patients without ACLD (non-ALCLD), (ii) patients with compensated ACLD (cACLD) but without CSPH, (iii) patients with cACLD and CSPH, (iv) patients with decompensated ACLD (decompensation prior to or within the first year after PBC diagnosis; dACLD). Thus, PBC-CSPH patients comprised cACLD with CSPH and all dACLD patients but did not include cACLD patients without CSPH.

Calculation of the cumulative incidence of decompensation as well as of the LTX-free survival for each CSPH specific clinical feature (as outlined above) was based on the period between PBC diagnosis and decompensation/death/LTX and whether patients were diagnosed with one of these characteristics during their course of disease or not. Importantly, patients with dACDL before or at baseline were not included into the calculation of the cumulative incidence of decompensation.

### Statistical analysis

Data assessment and statistical analysis was performed using IBM SPSS 26. Kolmogorov–Smirnov test was applied to distinguish between normally and non-normally distributed datasets. Mean and standard deviation as well as median and interquartile range were used whenever appropriate. Student t-test and Mann–Whitney-*U*-Test were applied to assess statistical significance for comparison of metric variables whereas chi-square-test was used for comparison of nominal variables. Graph-pad Prism 8 was utilized to compute Kaplan–Meier plots to illustrate survival. The start of follow-up was defined as the date of presentation/diagnosis of PBC at our clinic.

Survival after decompensation was only calculated in those patients who already had their first decompensating event at baseline. Survival rates were calculated between date of PBC diagnosis and death or liver transplantation. Odds ratios were calculated to estimate the impact of potential risk factors for development of decompensation, death or liver transplantation.

## Results

### PBC patient cohort (Table 1, Fig. 1)

**Table 1:** Patient characteristics

Patients	*N *= 333 (100%)
Age (median, IQR)	54.3 (45.7–64.2)
Women [*N* (%)]	289 (86.8%)
Liver Biopsy (*N* (%))	175 (52.6%)
AMA-M2(+) PBC	257 (77.2%)
PBC-ANA gp210	57 (17.1%)
PBC-ANA sp100	76 (22.8%)
Anti-centromere AB^¤^	42 (25.6%) ^¤^
AIH-Overlap [*N* (%)]^¥^	77 (23.1%)
Pruritus	96 (28.8%)
MELD [mean (SD)]	7.9 (± 3.0)
LSM [median (IQR; *N*)]^†^	7.2 kPa (5.5–13.2) kPa
HVPG [median (IQR; *N*)]^‡^	13 mmHg (7–21 mmHg)
ACLD at diagnosis	91 (27.3%)
cACLD without CSPH	33 (9.9%)
cACLD with CSPH	26 (7.8%)
dACLD (all with CSPH)	32 (9.6%)
Treatment with UDCA [*N* (%)]	301 (90.4%)
UDCA dose [mg/kg, median (IQR)]	13.4 (10.9–15.4)

	At presentation	Overall, during follow-up
Features of CSPH	58/333 (17.4%)	127/333 (38.1%)
Splenomegaly	49/333 (14.7%)	98/333 (29.4%)
Portosystemic collaterals	26/333 (7.8%)	62/333 (18.6%)
Gastroesophageal varices	28/333 (8.4%)	63/333 (18.6)
HVPG ≥ 10 mmHg	10/333 (3.0%)	22/333 (6.6%)
Ascites	32/333 (9.6%)	62/333 (18.6%)
Variceal bleeding	8/333 (2.4%)	20/333 (6.0%)
Hepatic encephalopathy	14/333 (4.2%)	22/333 (6.6%)
Time between PBC diagnosis and CSPH (months, mea*N *± SD)	10.6 ± 7.15	
Features of CSPH before PBC diagnosis	50/333 (15.0%)	
Median follow-up (years, median (IQR))	5.8 (2.7–12.6)	

CSPH-related decompensation	At presentation	Overall, during follow-up
Any decompensating event	32/58 (55.2%)	72/127 (56.7%)
Ascites	32/58 (55.2%)	62/127 (48.8%)
Variceal bleeding	8/58 (13.8%)	20/127 (15.7%)
Hepatic encephalopathy	14/58 (24.1%)	22/127 (17.3%)
SBP	2/58 (3.4%)	4/127 (3.1%)
HRS	11/58 (19.0%)	21/127 (16.5%)
CSPH-treatment and outcomes		
NSBB therapy	65/333 (19.5%)	
NSBB prescription in CSPH patients	25/58 (43.1%)	55/127 (43.3%)
NSBB prescription in dACLD patients	20/32 (62.5%)	43/72 (60%)
EBL therapy	17/58 (29.3%)	36/127 (28.3%)
TIPS	2/58 (3.4%)	7/127 (5.5%)
OLT	8/58 (13.8%)	14/127 (11.0%)
Death	16/58 (27.6)	44/127 (34.6%)
CSPH-related death	16/58 (27.6%)	18/127 (14.1%)

Abbreviations: please refer to the abbreviations section above

^†^LSM by TE was performed in 217 patients

^‡^HVPG measurements were performed in 35 patients

^¥^Patients with PBC-AIH overlap syndrome were diagnosed according to the Paris criteria [39]

^¤^Anti-centromere status was available in *N *= 164 patients

Our final study population included 333 patients. For further details concerning demographic characteristics, prevalence of ACLD and features of CSPH at presentation as well as targeted treatment of portal hypertension please refer to Table 1 and Fig. 1.

### Development of ACLD and CSPH during follow-up (Table 1, Fig. 1)

During a median follow-up duration of 5.8 (IQR 2.7–12.6) years, 168 (50.5%) patients presented features suggestive of ACLD, of whom 41 (12.3%) remained without features of CSPH during further follow-up. 70 (21.0%) developed ACLD with features of CSPH, of whom 55 (16.5%) remained compensated throughout the observational period. All in all, 72 (21.6%) patients developed dACLD, resulting in a total of 127 (55 + 72) (38.1%) patients, identified with at least one feature of CSPH.

During follow-up, splenomegaly was the most frequent CSPH-related feature, affecting 98 (29.4%) patients, whereas portosystemic collaterals were found in 62 (18.6%) and esophageal varices in 63 (18.6%) patients, respectively.

62 (18.6%) patients of our PBC cohort developed ascites, the most frequent decompensating event during follow-up. Acute variceal bleeding and hepatic encephalopathy occurred in 20 (6.0%) and 22 (6.6%) PBC patients, respectively. Overall, 18 of 127 PBC patients with CSPH (14.1%) died due to decompensating events.

### Rate of CSPH-related decompensating events (Table 2, Fig. 2)

**Table 2:** Cumulative incidence of hepatic decompensation (i.e. dACLD) during follow-up according to distinct characteristics at clinical presentation

Variable	Cumulative incidence of dACLD			
	1–3 Years	5 Years	10 Years	*N* total†
Compensated patients (non-ACLD, cACLD)	2.8%	5.0%	11.4%	37/301
Normal PLT and LSM < 15 kPa	0.0%	0.0%	5.2%	6/160
Thrombocytopenia (< 150G/L) or LSM ≥ 15 kPa	8.7%	16%	27.9%	19/51
No ACLD^‡^	1.9%	4.5%	11.0%	32/242
cACLD without CSPH^‡^	4.3%	4.3%	21.5%	3/33
cACLD with CSPH^‡^	13.3%	13.3%	13.3%	2/26
Any CSPH (cACLD + dACLD) at baseline^¥^	18.4%	26.8%	41.4%	8/58
Splenomegaly	6.3%	15.0%	29.9%	24/73
Esophageal varices	10.1%	20.9%	37.5%	26/41
Portosystemic collaterals	7.4%	17.8%	36.9%	26/41

Risk of hepatic decompensation according to distinct clinical characteristics within 1–3 years, 5 years and 10 years of follow-up

^†^Number of patients with at least one decompensating event/number of patients included in this analysis

^‡^Subgroup classification according to baseline characteristics

^¥^Baseline decompensation events were not considered

**Fig. 2:**
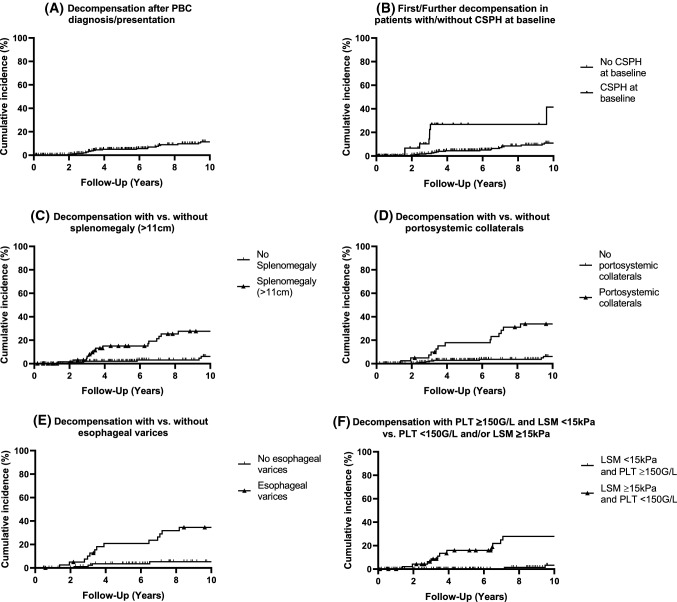
Decompensation rates in PBC patients according to CSPH features*. A 10-year cumulative incidence of dACLD among all PBC patients, who were compensated at baseline; *N *= 21/301 (*N *= event/total). B 10-year cumulative incidence of first or further decompensation in patients with CSPH at baseline (*N *= 8/58) (*N *= 8/58; log-rank *P *< 0.0001) vs. without CSPH at baseline (*N *= 20/275). C 10-year cumulative incidence of dACLD in compensated PBC patients with splenomegaly (*N *= 16/73; log-rank *P *< 0.001) vs. without splenomegaly (*N *= 6/169). D 10-year cumulative incidence of dACLD in compensated patients with portosystemic collaterals (*N *= 14/41; log-rank *P *< 0.0001) vs. without portosystemic collaterals (*N *= 8/213). E 10-year cumulative incidence of dACLD in patients with esophageal varices (*N *= 14/41; log-rank *P *< 0.0001) vs patients without esophageal varices (*N *= 4/95). F 10-year cumulative incidence of dACLD in patients with normal platelet count (≥ 150G/L) and LSM < 15 kPa (*N *= 3/160) versus patients with either thrombopenia (< 150G/L) and/or LSM ≥ 15 kPa (*N *= 11/51; log-rank *P *< 0.001). *The number in brackets (*N *= *x*/*y*) shows the number of patients who progressed to dACLD within 10-years (*x*) in relation to the number of patients who met a distinct criterion (e.g., splenomegaly) and were included in this analysis (*y*). Divergence with Table 1 is possible since we excluded patients with dACLD previous or at the time of baseline from this analysis; excluding (B)

Among initially compensated PBC patients the 3 year decompensation rate was 2.8%, whereas patients without ACLD at diagnosis showed a 1.9% 3 year decompensation rate. Contrastingly, patients with cACLD but without CSPH and cACLD with CSPH had 3 year decompensation rates of 4.3% and 13.3%, respectively.

Esophageal varices during gastroscopy and portosystemic collaterals on radiographic imaging showed similarly high 3 and 10 year decompensation-rates (10.1% and 37.5% vs. 7.4% and 36.9% respectively), whereas splenomegaly revealed a comparably low 3 and 10 year decompensation rate (6.3% and 29.9%).

### Survival of PBC patients  according to CSPH features (Table 3, Figs. 3, 4)

**Table 3:** Transplant-free survival according to distinct characteristics of patients with PBC

Variable	Liver transplant-free survival				
	1 Year	3 Years	5 Years	10 Years	*N* ^†^ total
Overall survival	95.2%	93.5%	90.1%	83.1%	54/333
Normal PLT count and LSM < 15 kPa	100.0%	100.0%	100.0%	100.0%	2/163
Thrombocytopenia (< 150G/L) or LSM ≥ 15 kPa	84.4%	79.6%	75.8%	68.2%	26/73
No ACLD	98.7%	98.7%	96.6%	90.8%	29/242
cACLD without CSPH	96.9%	96.9%	96.9%	77.7%	3/33
cACLD with CSPH	90.9%	79.7%	57.4%	57.4%	6/26
dACLD	69.1%	48.5%	36.4%	24.3%	16/32
Any CSPH (cACLD + dACLD) at baseline	79.2%	66.3%	47.7%	35.8%	22/58
Splenomegaly	88.4%	83.7%	74.2%	67.0%	32/98
Portosystemic collaterals	87.0%	80.2%	70.1%	58.2%	31/62
Esophageal varices	88.8%	81.7%	71.2%	61.4%	29/63
Ascites	84.8%	75.4%	66.3%	52.6%	29/62

Survival according to baseline characteristics at 1, 3, 5 and 10 years of follow-up

^†^Number of patients who underwent liver transplantation or died/number of patients included in this analysis

**Fig. 3:**
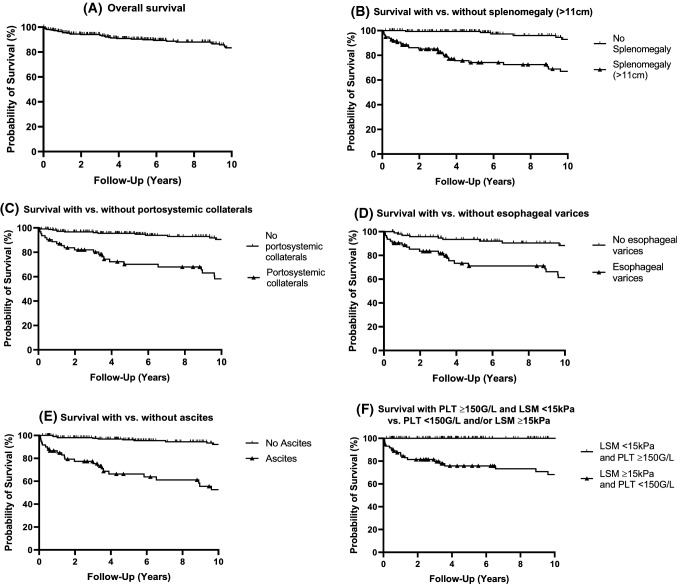
LTX-free survival in PBC patients. A 10-year cumulative survival in all PBC patients (*N *= 38/333). B 10-year cumulative survival in patients with splenomegaly (*N *= 26/98; log-rank *P *< 0.0001) vs. without splenomegaly (*N *= 6/175). C 1-year cumulative survival in patients with portosystemic collaterals (*N *= 22/62; log-rank *P *< 0.0001) vs. without portosystemic collaterals (*N *= 14/224). D 10-year cumulative survival in patients with esophageal varices (*N *= 20/63; log-rank *P *< 0.0001) vs. without esophageal varices (*N *= 9/101). E 10-year cumulative survival in patients with ascites (*N *= 23/62; log-rank *P *< 0.0001) vs. without ascites (*N *= 11/217). F 10-year cumulative survival in patients with normal platelet count (≥ 150G/L) and LSM < 15 kPa (*N *= 0/163) and patients with thrombocytopenia (< 150G/L) and/or LSM ≥ 15 kPa (*N *= 19/73; log-rank *P *< 0.0001)

**Fig. 4:**
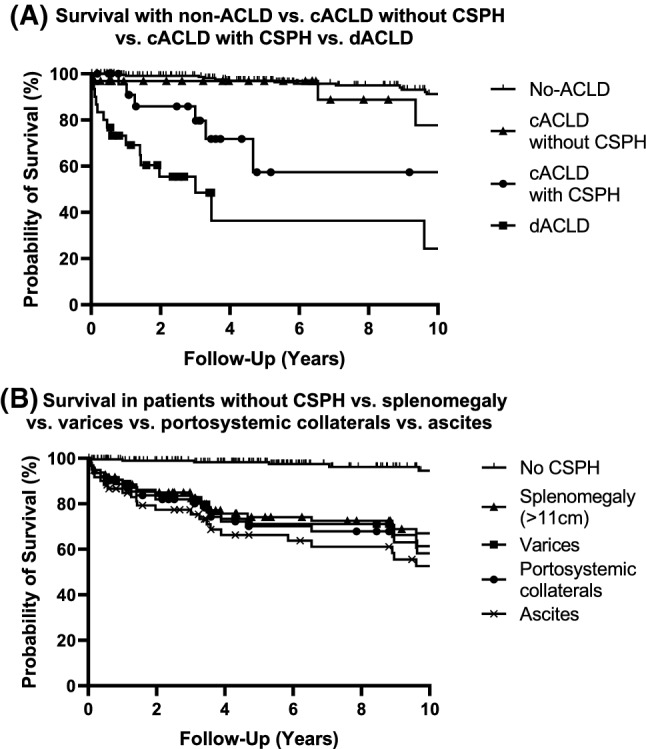
LTX-free survival in PBC patients according to ACLD and CSPH status. A 10 year cumulative survival in non-ACLD (*N *= 14/241) vs. cACLD without CSPH (*N *= 3/33) vs. cACLD and CSPH (*N *= 6/26) vs. dACLD (*N *= 15/32). There was no difference in TFS between non-ACLD and cACLD w/o CSPH (log-rank *P *= 0.384), while TFS gradually decreased in cACLD with CSPH (log-rank test vs. non-ACLD *P *= 0.025 and vs. ACLD-w/o CSPH *P *< 0.001) and in dACLD (log-rank test vs. cACLD-with CSPH *P *= 0.019, vs. non-ACLD and cACLD-w/o CSPH *P *< 0.001). B 10-year cumulative survival in patients without CSPH (*N *= 7/205) vs. patients with splenomegaly (*N *= 26/98) vs. patients with esophageal varices (*N *= 20/63) vs. patients with portosystemic collaterals (*N *= 22/62) vs. patients with ascites (*N *= 23/62). There was no significant difference in TFS between splenomegaly and ascites (log-rank *P *= 0.0569) as well as between splenomegaly, portosystemic collaterals (log-rank *P *= 0.596) and esophageal varices (log-rank *P *= 0.405). However, TFS differed significantly between patients without CSPH and splenomegaly/portosystemic collaterals/esophageal varices/ascites (log-rank *P *< 0.0001)

During a median follow-up of 5.8 years (IQR 2.7–12.6 years), 44 (13.21%) PBC patients died, including 23 liver-related deaths of which 18 were attributed to CSPH-related complications. PBC patients with CSPH features had a liver related mortality of 17.3% (22/127) and a 7.2-fold risk of death compared to those without CSPH.

10 year survival of our entire PBC cohort was 83.1%. PBC patients without ACLD had similar survival rates at 1, 3 and 5 years, when compared to PBC patients with cACLD without CSPH (log-rank *P *= 0.384) (FFI Table 3, Figs. 3, 4).

Survival rates in patients with cACLD and CSPH were significantly worse when compared to patients without ACLD (log-rank *P *< 0.001) or to patients with cACLD without CSPH (log rank *P *= 0.025). In comparison to other stages of ACLD, patients with dACLD showed the lowest one year and 10 year survival rates (69.9% and 24.3%, respectively; log-rank.

*P *≤ 0.019).

Patients diagnosed with splenomegaly had similar one-year survival rates compared to patients with esophageal varices or portosystemic collaterals (FFI Table 3, Fig. 4), whereas 10 year survival rates ranged from 67.0% to 61.4% and 58.2%, respectively. Patients diagnosed with ascites had the poorest one-year and 10 year survival rates (84.8% and 52.6% respectively).

### Prognostic significance of LSM and platelet count (Tables 2, 3, Fig. 3)

Among PBC patients with both normal platelet count (≥ 150 G/L) and LSM < 15 kPa at transient elastography (*N *= 160), the 10 year cumulative decompensation rate was only 5.2%. In contrast, patients presenting with either thrombocytopenia (< 150 G/L) or LSM ≥ 15 kPa at baseline (*N *= 39) had a cumulative decompensation rate of 8.7% after 3 years and of 27.9% after 10 years of follow-up.

Patients matching at least one of the criteria (thrombocytopenia (< 150 G/L) or LSM ≥ 15 kPa) had a 13.7-fold increased risk to develop CSPH during follow-up as compared to patients with a normal platelet count and LSM < 15 kPa.

Patients with both a normal platelet count and LSM < 15 kPa had a 100% survival rate after 10 years, whereas PBC patients presenting with thrombocytopenia and/or LSM ≥ 15 kPa showed a 10 year survival rate of only 68.2%.

## Discussion

In our PBC cohort, 10 year overall survival was 83.1% and therefore similar to recent data presented by Tanaka et al. [23] *(88% 10 year survival in UDCA treated patients)*.

Comparison between patients with splenomegaly, portosystemic collaterals, and esophageal varices (FFI Tables 2, 3) confirms that each CSPH-related feature is linked to a different probability of subsequent decompensation and survival [1, 24]. The same goes for stratifying patients according to their ACLD stage (FFI Tables 2, 3). Hence, screening for features of CSPH in patients with PBC is crucial as it allows for early treatment intensification and individualized risk stratification.

The specific PBC target group for CSPH screening includes patients with compensated advanced chronic liver disease (cACLD), which can be suspected by LSM ≥ 15 kPa [25]. The concept of cACLD, introduced at Baveno VI [25], primarily based on patients with viral hepatitis C, but Moctezuma-Velazquez et al*.* already confirmed the applicability of Baveno VI (LSM ≥ 20 kPa and PLT < 150 G/l) and extended Baveno VI criteria (LSM ≥ 25 kPa and PLT < 150G/L) to predict the presence of esophageal varices requiring treatment in patients with PBC [26].

In this study we extended and modified the Baveno VI criteria to predict subsequent decompensation and transplant free survival. For this purpose, we decreased the LSM cutoff to ≥ 15 kPa which is supported by recent data showing that a LSM cutoff at ≥ 14.4 kPa has a high accuracy predicting F4 fibrosis and subsequent decompensation in patients with PBC [27].

Patients matching at least one of both criteria, LSM ≥ 15 kPa and PLT < 150 G/l, had a significantly increased risk for subsequent decompensation and death as opposed to those with LSM < 15 kPa and normal platelet count (FFI Tables 2, 3). This supports the use of (repeated) LSM [20, 21] and platelet counts in patients with PBC as both fit easily into clinical routine and hold considerable prognostic value for risk stratification.

Evaluation of PH-targeted treatment revealed that only 43.3% of those with CSPH and 60.0% of those with dACLD received NSBBs. These results indicate a significant undertreatment of CSPH in PBC patients, especially in consideration of recent studies [28], which observed a decreased risk for decompensation and mortality in CSPH-patients treated with NSBBs [29–31]. While a considerable number of PBC patients developed ascites (*N *= 62) and variceal bleeding (*N *= 20), TIPS—as a highly effective intervention to control ascites [31] and severe variceal bleeding [33]—was only used in 7 patients. Since 40.9% of all deaths observed in our PBC cohort were CSPH-related, the use of TIPS for treatment of severe CSPH complications should be encouraged, as TIPS reduces mortality in the setting of refractory ascites [32, 34] as well as in high-risk variceal bleeding [33, 35]. Overall *N *= 72 (21.6%) progressed to dACLD during their course of disease which confirms results from previous literature (24.6%) [36].

28.8% of all patients suffered from pruritus which is experienced as a significant reduction in Quality of Life [37]. However, similar to previous studies [37, 38] the odds towards development of dACLD were not increased (QR 0.86; *P *= 0.606) in PBC patients with pruritus.

Prevalence of PBC-AIH-Overlap, which was diagnosed according to the PARIS criteria [39], was considerably higher in our cohort when compared to preexisting literature (3–10%) [40, 41]. Recruitment of our collective of patients at the Medical University of Vienna, a tertiary care hospital, may have caused a selection bias towards a higher single-center prevalence of PBC-AIH Overlap Syndrome. All patients with PBC-AIH Overlap received state of the art treatment with UDCA augmented by steroids and immunosupressants [2]. Optimized therapy and regularly scheduled control visits may therefore explain why patients with AIH did not show increased odds regarding decompensation within our cohort (OR 0.937; *P *= 0.838; Suppl. Table 1).

A relevant limitation of this study is its retrospective design, which impeded a comprehensive data acquisition and resulted in a heterogenous follow-up. Hence, Kaplan–Meier plots were used to estimate survival and the cumulative incidence of decompensation. Furthermore, selection bias towards patients with more advanced PBC disease is likely since our center is a tertiary care referral center for ACLD (of any etiology). For the same reason, we might have underestimated overall survival. Nonetheless our study population is representative for PBC patients of other tertiary care and academic centers [23]. Unfortunately, we could not address the specific impact of PBC treatment nor differentiate between UDCA responders vs. non-responders. However, as evident by the high rate of UDCA use at recommended doses, we assume that most CSPH-related complications have occurred despite UDCA therapy.

The inclusion of splenomegaly as criterion for CSPH is controversially discussed as body height and CSPH-unrelated factors such as immune dysregulation have been reported to impact on spleen size [42, 43]. Splenomegaly, however, is a widely accepted and frequent [44] clinical feature, that warrants further examination towards CSPH if detected during routine imaging. Moreover, Jung et al. [45] described splenomegaly as a “sum score” in primary sclerosing cholangitis (PSC), reflecting different pathophysiological events, including CSPH [45]. Their data additionally indicated a significantly impaired prognosis of PSC patients with increasing spleen-diameter.

We are aware that ascites and hepatic encephalopathy are not part of the initially proposed Baveno VI guidelines either. We nonetheless decided to include these parameters in our definition of CSPH since both have been associated with portal hypertension [46–48] and regularly occur during end-stage chronic liver disease.

The strength of this study is the individual assessment of different CSPH-related features, allowing clinicians to make prognostic estimations that support the planning of follow-up visits and facilitate decision-making regarding screening and treatment of CSPH-related complications. Importantly, our study confirms that risk stratification of patients with PBC according to different stages of ACLD is predictive regarding decompensation and transplant free survival. We further confirmed that the simple combination of LSM and platelet count, as a readily available score, is of excellent prognostic value in the setting of PBC. This allows for individualized care and may support early treatment intensification.

In conclusion, CSPH develops in a considerable proportion of PBC patients. Splenomegaly was the most frequent sign of CSPH and ascites the most frequent first decompensating event. The combination of LSM ≥ 15 kPa and/or thrombocytopenia (< 150G/L) represents a valuable non-invasive risk score for CSPH-related decompensation and mortality in patients with PBC. Clinicians should regularly monitor PBC patients for distinct features of CSPH, such as splenomegaly or portosystemic collaterals as they may occur earlier than varices at endoscopy but already indicate an impaired prognosis. Considering that as many as 40.9% of all deaths in our PBC cohort were caused by CSPH-related complications, the use of NSBB and TIPS for treatment of CSPH should be encouraged.

Future studies should evaluate if CSPH screening and early initiation of CSPH-targeted therapies improve prognosis and survival in patients with PBC.

## Supplementary Information

Below is the link to the electronic supplementary material.

Supplementary file1 (DOCX 15 KB)

## Data Availability

Data, analytic methods, and study materials will be made available upon request by the corresponding author.

## Abbreviations

ACLD: Advanced chronic liver disease
AIH: Autoimmune hepatitis
cACLD: Compensated ACLD
CSPH: Clinically significant portal hypertension
dACLD: Decompensated ACLD
EBL: Endoscopic band ligation
FFI: For further information
HRS: Hepato-renal syndrome
HVPG: Hepatic venous pressure gradient
IQR: Inter quartile range
LSM: Liver stiffness measurement
MELD-Score: Model of End Stage Liver Disease-Score
OLT: Orthotopic liver transplantation
PLT: Platelet count
SBP: Spontaneous bacterial peritonitis
SD: Standard deviation
TE: Transient elastography
TFS: Transplant free survival
TIPS: Transjugular intrahepatic portosystemic shunt
UDCA: Ursodesoxycholic acid
w/o: Without
