# Supplementary material for: Distinct prognostic value of different portal hypertension-associated features in patients with primary biliary cholangitis
Source: J Gastroenterol. 2021 Dec 11;57(2):99–110. doi: 10.1007/s00535-021-01839-3 (PMC8831368; doi:10.1007/s00535-021-01839-3)
Supplement: Supplementary file 1 — Supplementary file1 (DOCX 15 KB) [file 535_2021_1839_MOESM1_ESM.docx]

**Suppl. Table-ST1.: Impact of clinical and serological risk factors on disease progression**

| **Stage of disease** | **Any sign of CSPH** | | | | **cACLD** | | | | **dACLD** | | | |
| --- | --- | --- | --- | --- | --- | --- | --- | --- | --- | --- | --- | --- |
| *Variable* | *Present* | *Absent* | *OR* | *p* | *Present* | *Absent* | *OR* | *p* | *Present* | *Absent* | *OR* | *p* |
| AIH-Overlap | 40.3% | 37.5% | 1.12 | *0.662* | 27.3% | 23.6% | 1.21 | *0.547* | 20.8% | 21.9% | 0.94 | *0.838* |
| Pruritus | 39.6% | 37.6% | 1.09 | *0.730* | 28.6% | 22.8% | 1.36 | *0.299* | 19.8% | 22.4% | 0.86 | *0.606* |
| SP100 positive | 34.2% | 32.2% | 1.09 | *0.774* | 23.1% | 21.1% | 1.12 | *0.760* | 18.4% | 15.7% | 1.21 | *0.619* |
| GP210 positive | 49.1% | 30.4% | 2.21 | ***0.013*** | 30.4% | 20.5% | 1.7 | *0.173* | 33.3% | 15.2% | 2.79 | ***0.004*** |
| AMA-M2 positive | 37.0% | 37.7% | 0.97 | *0.916* | 23.1% | 27.7% | 0.78 | *0.503* | 21.0% | 18.9% | 1.14 | *0.726* |
| Anti-centromere | 47.6% | 37.7% | 1.5 | 0.258 | 31.3% | 22.5% | 1.56 | 0.319 | 28.6% | 22.1% | 1.41 | 0.398 |
| ANA | 38.6% | 34.2% | 1.21 | 0.418 | 27.1% | 19.9% | 1.5 | 0.159 | 19.9% | 20.5% | 0.91 | 0.731 |

**Supplementary Table-ST1.**

Illustration of AIH-Overlap status, pruritus and autoantibody serology and the respective impact on the development of CSPH, cACLD and dACLD. OR: Odds ratio.
